# Proteoliposome Preparation and Functional Reconstitution of Human Mitochondrial Transporters Via Wheat Germ Cell‐Free Expression System

**DOI:** 10.1002/cpz1.70500

**Published:** 2026-10-26

**Authors:** Davood Kharaghani, Haotian Deng, Xiaoyang Su

**Affiliations:** ^1^ Department of Medicine, Rutgers‐Robert Wood Johnson Medical School Rutgers University New Brunswick New Jersey

**Keywords:** cell‐free expression, membrane protein, mitochondria, proteoliposome, SLC transporters, solute carriers, wheat germ

## Abstract

Mitochondrial membrane proteins play a crucial role in the transport of metabolites essential to cellular metabolism, and their dysfunction is linked to a wide range of metabolic diseases. Here, we present a method for the expression, reconstitution, and analysis of members of the solute carrier family 25 (SLC25), the largest family of human mitochondrial transport proteins, using the wheat germ cell‐free protein synthesis (WG‐CFPS) system. This approach enables the generation of proteoliposomes containing a defined and highly purified protein species. This workflow enables efficient production of active membrane proteins without the limitations of a cell‐based expression system, such as aggregation into inclusion bodies, the need for purification, and the cytotoxic effects of membrane protein overexpression. SLC25A11 and SLC25A21 proteins, as examples of human mitochondrial transporters, are directly translated into asolectin liposomes in the presence of the non‐ionic detergent Brij‐35. Later, to form proteoliposomes suitable for downstream transport assays, the proteins were reconstituted into a substrate‐loaded liposome. The article provides detailed procedures for template preparation, cell‐free expression, proteoliposome assembly, and validation of incorporated protein transport activity. This method, by reducing the time required to express, purify, and reconstitute membrane proteins, provides a platform for studying human mitochondrial metabolite transport activity. Once the reagents and plasmid are prepared, the complete work, from protein expression to functional analysis, can be completed within 3 days. © 2026 The Author(s). *Current Protocols* published by Wiley Periodicals LLC.

**Basic Protocol 1**: Plasmid design and development

**Basic Protocol 2**: Cell‐free protein expression

**Basic Protocol 3**: Active reconstitution and evaluation of transport activity

## INTRODUCTION

Mitochondrial metabolite transport is a fundamental process that underpins cellular energy homeostasis and influences the pathogenesis of numerous metabolic diseases (Anselme et al., 2025; Cunningham & Rutter, 2020; Xie et al., 2025). The major challenge in defining substrates and inhibitors for mitochondrial carrier studies is the availability of an assay that can measure metabolite transport through mitochondrial transporters with reasonable throughput (Cunningham & Rutter, 2020). While whole‐cell uptake assays or metabolomic profiling provide broad insights into nutrient utilization, they often lack the compartmental resolution required to isolate mitochondrial transport from plasma membrane flux and cytosolic metabolism. Accurate measurement of carrier function thus necessitates either the labor‐intensive purification of intact mitochondria or the use of reconstituted systems. Since the first demonstration that mitochondrial transport proteins of the solute carrier family 25 (SLC25) can be purified and functionally reconstituted into liposomes to dissect substrate transport and exchange activity (Shertzer & Racker, 1976), biochemical reconstitution has served as an essential tool for elucidating mitochondrial metabolite transport (Atlante & Valenti, 2023).

In recent years, proteoliposomes have greatly facilitated the study of membrane proteins (Colson et al., 2025) by enabling the assessment of metabolite transportation (Banerjee & Datta, 1983). However, the traditional cell‐based method for overexpression, purification, and reconstitution of membrane proteins into liposomes is time‐consuming and costly (Manzer et al., 2023).

Although WG‐CFPS‐based expression and Brij‐35‐assisted co‐translational reconstitution into liposomes have been previously established, these approaches have primarily been developed and applied to membrane proteins and plant transporters. The methodological contribution of the present work is the adaptation and integration of this strategy into a streamlined workflow specifically designed to functionally characterize human mitochondrial SLC25 carriers. To address this gap, we present a proteoliposome‐based workflow for the in vitro expression, reconstitution, and characterization of mitochondrial transport proteins’ transport activity. Therefore, examples of human mitochondrial carriers were tested, including solute carrier family 25 member 11 (SLC25A11) and solute carrier family 25 member 21 (SLC25A21). This method uses the wheat germ cell‐free protein synthesis (WG‐CFPS) system in the presence of Brij‐35 to enhance the solubility of membrane proteins, thereby promoting proper folding and preserving their transport activity (Nozawa et al., 2007). Finally, the transport activity of reconstituted SLC25A11 and SLC25A21 was evaluated using radioactive isotope‐labeled tracer.

The activity assay of mitochondrial transport proteins is technically demanding because when expressed in living cells (Ardehali et al., 2005; Carpenter et al., 2008). Conventional cell‐based expression and purification typically require weeks to complete (Katzen et al., 2009; Zemella et al., 2015). These limitations significantly restrict systematic and comparative studies of mitochondrial carriers and disease‐associated variants.

A central challenge in transport protein reconstitution is maintaining protein solubility while preserving native conformation and transport activity (Zhou et al., 2025). This is addressed by incorporating the mild nonionic detergent Brij‐35 directly into the WG‐CFPS reaction (Nozawa et al., 2007), which is compatible with downstream liposome assembly and transport activity assay.

For protein translation, the WG‐CFPS system, translation, and reconstitution strategy avoids protein isolation steps and heterogeneous protein populations that often compromise activity and significantly reduce total experimental time (Nozawa, Fujimoto, et al., 2011).

While traditional in vivo protein expression systems, such as *Escherichia coli* or HEK293, require the target protein to be non‐toxic and isolated from a highly complex cellular environment, the WG‐CFPS provides significantly cleaner starting materials, streamlining the purification of FLAG‐ or HA‐tagged proteins (Aceti et al., 2015). In living cell lysates, purification efficiency is often compromised by the presence of thousands of endogenous host proteins and high concentrations of genomic DNA and RNA, which increase lysate viscosity and promote non‐specific binding to the affinity resin (Didovyk et al., 2017). This complexity often leads to persistent background noise and requires multiple wash steps to achieve acceptable purity.

In contrast, WG‐CFPS coupled with Brij‐35 detergent‐assisted co‐reconstitution (Nozawa et al., 2007), enabling co‐translational membrane insertion under mild detergent conditions. WG‐CFPS has been increasingly recognized for its efficiency in membrane protein translation and reconstitution, offering advantages over the cell‐based system (Jiang et al., 2023), where hydrophobic mitochondrial carriers frequently form β‐rich inclusion bodies that require aggressive denaturation/refolding (Jorrín‐Novo et al., 2014).

## STRATEGIC PLANNING

This article provides instructions for performing our transporter translation‐reconstitution‐activity pipeline, originally developed for plant proteins in the context of the WG‐CFPS (Bernhardt et al., 2012; Nozawa, Fujimoto, et al., 2011; Nozawa, Ogasawara, et al., 2011; Nozawa et al., 2020). Here, we used WG‐CFPS, coupled with Brij‐35‐assisted detergent in vitro translation, followed by reconstitution, to analyze the transport activity of human mitochondrial carriers of the SLC25 family. The workflow is divided into three key phases: (i) plasmid design and development, (ii) cell‐free protein expression, and (iii) active reconstitution and evaluation of transport activity. Splitting the pipeline enhances efficiency by using only the correct cloned plasmids from Phase 1 to express the correct protein in Phase 2, resulting in membrane‐competent proteoliposomes that proceed to full activity testing in Phase 3. Because the carrier function requires a membrane environment and oligomeric packing that support conformational exchange (Nozawa & Tozawa, 2014), we prioritize in vitro translation into lipid bilayers, coupled with mild non‐ionic detergent. Membrane transporters, particularly mitochondrial carriers, require proper expression, lipid interactions, and oligomeric assembly (Wagner et al., 2006) to support the conformational exchange necessary for substrate translocation. Direct progression from expression to functional assays without intermediate quality control often results in a low success rate and ambiguous negative results (Nozawa & Tozawa, 2014). Therefore, this protocol incorporates an intermediate screening phase that advances only constructs demonstrating adequate expression in the lipid bilayer and membrane competence.

### Basic Protocol 1

In the first phase of this article, a plasmid carrying the gene of interest was designed and generated, as shown in Figure 1.

**Figure 1:**
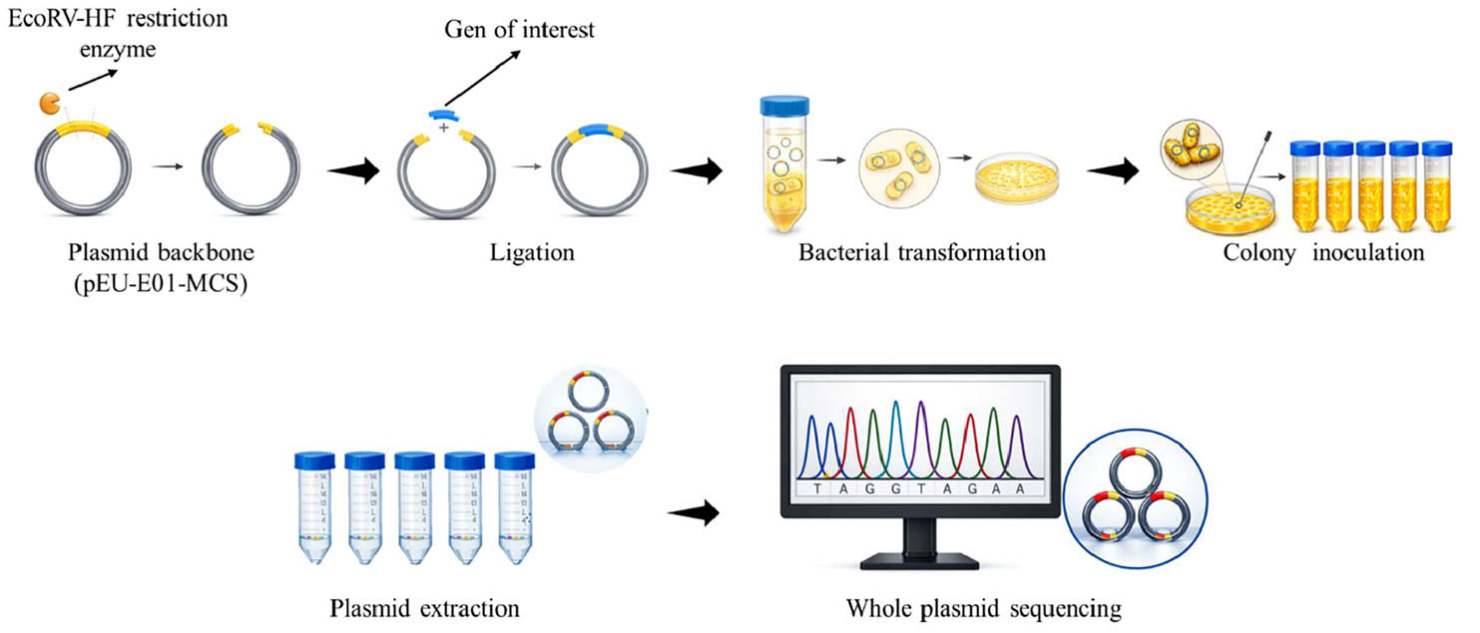
Cloning and validation workflow. The plasmid backbone was digested with EcoRV, and the gene was inserted using Gibson assembly. The assembled plasmid was transformed into bacteria, followed by colony selection and culture. Plasmids were purified and verified by whole‐plasmid sequencing.

First, the vector plasmid pEU‐E01‐MCS, which features a bacteriophage SP6 promoter and the E01 translational enhancer, was selected (Boyer et al., 2024). Cell‐free translation systems can synthesize protein at high speed and accuracy using the expression vector pEU, which carries the respective gene under the control of the SP6 RNA polymerase promoter (Sawasaki et al., 2000). The coding sequence for the gene of interest can be acquired either by amplification from cDNA or by synthesis. To facilitate the Gibson assembly, the coding sequence is flanked by adapter sequences: AACCACCTATCTACATCACCAAGAT (at 5’) and GGTACCGAGCTCGAGAACTAGTGAT (at 3’) (see Supporting Information, 1.1. Assembly and Sequences, Supplementary Table 1). The vector is digested with EcoRV, and the coding sequence is assembled into the plasmid backbone using New England Biolabs HiFi Assembly following the manufacturer's instructions.

Next, the assembled DNA was used to chemically transform competent *E. coli* cells (Froger & Hall, 2007). Transformed cells were plated on selective agar containing 100 µg/ml ampicillin and grown overnight at 37°C. The next day, the colonies were screened by colony PCR, and the colonies with insert verification were sent for sequencing. Phase 1 necessitates the use of the following components:

(1) A pEU‐E01‐MCS plasmid backbone with an appropriate replication origin and antibiotic selection marker.
(2) The insert DNA sequence encoding the gene of interest.
(3) Chemically competent *E. coli* cells (DH5α or equivalent).
(4) Regents for DNA assembly, transformation, selection, and plasmid extraction (e.g., antibiotic, culture medium, Midi‐prep kit).

#### Experimental control

Using a plasmid with a previously validated insert is not obligatory, but it can serve as a useful positive control to ensure that the transformation and extraction procedures are functioning correctly. As a negative control, an empty plasmid backbone (lacking the gene of interest) can be used to evaluate successful insertion. In all cases, successful plasmid extraction was verified by confirming sequence identity and integrity using colony PCR and whole‐plasmid sequencing, followed by multiple sequence alignment. Plasmid concentration was determined spectrophotometrically to ensure consistency across experiments.

### Basic Protocol 2

In the second phase of this article, the focus shifts to in vitro protein translation and membrane insertion competence, as shown in Figure 2. This phase utilizes Brij‐35‐containing WG‐CFPS, combined with a simple liposome co‐migration, to assess the proper incorporation of the protein of interest into lipid bilayers (Nozawa et al., 2007; Nozawa & Tozawa, 2014).

**Figure 2:**
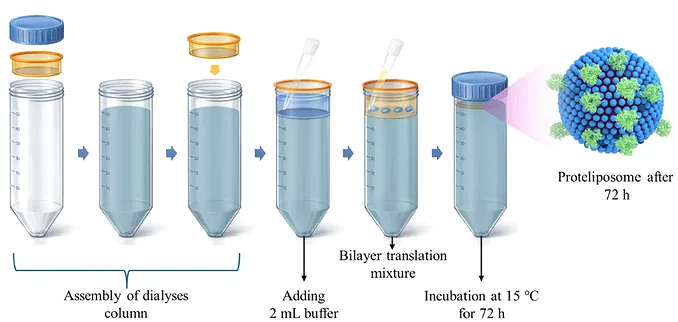
Proteoliposome reconstitution workflow. Stepwise schematic illustration of dialysis column and incorporation of the bilayer translation mixture assembly leading to the formation of a proteoliposome.

The gene of interest, cloned in phase 1, was expressed in a WG‐CFPS in the presence of asolectin liposomes. To enhance solubility and proper folding, the protein was translated in the presence of Brij‐35 and asolectin liposomes during incubation (Nozawa et al., 2007). The mixture is then subjected to simple centrifugation and washing steps, allowing proteins successfully embedded in liposomes to sediment while unincorporated proteins, such as wheat germ ribosomal proteins, are removed. Phase 2 necessitates the use of the following components:

(1) Plasmid DNA encoding the gene of interest (from Phase 1).
(2) WG‐CFPS expression reagent and equipment.
(3) Brij‐35 to increase solubility and promote proper membrane insertion.

#### Experimental control

As a negative control, protein‐free liposomes are used to assess nonspecific co‐migration. To ensure that both control and experimental proteoliposomes have comparable incorporation and solubility, total protein levels are measured using the BSA assay kit.

### Basic Protocol 3

In the third phase of this article, proteins embedded with asolectin from Phase 2 are reconstituted into preloaded liposomes containing the metabolite of interest, as shown in Figure 3.

**Figure 3:**
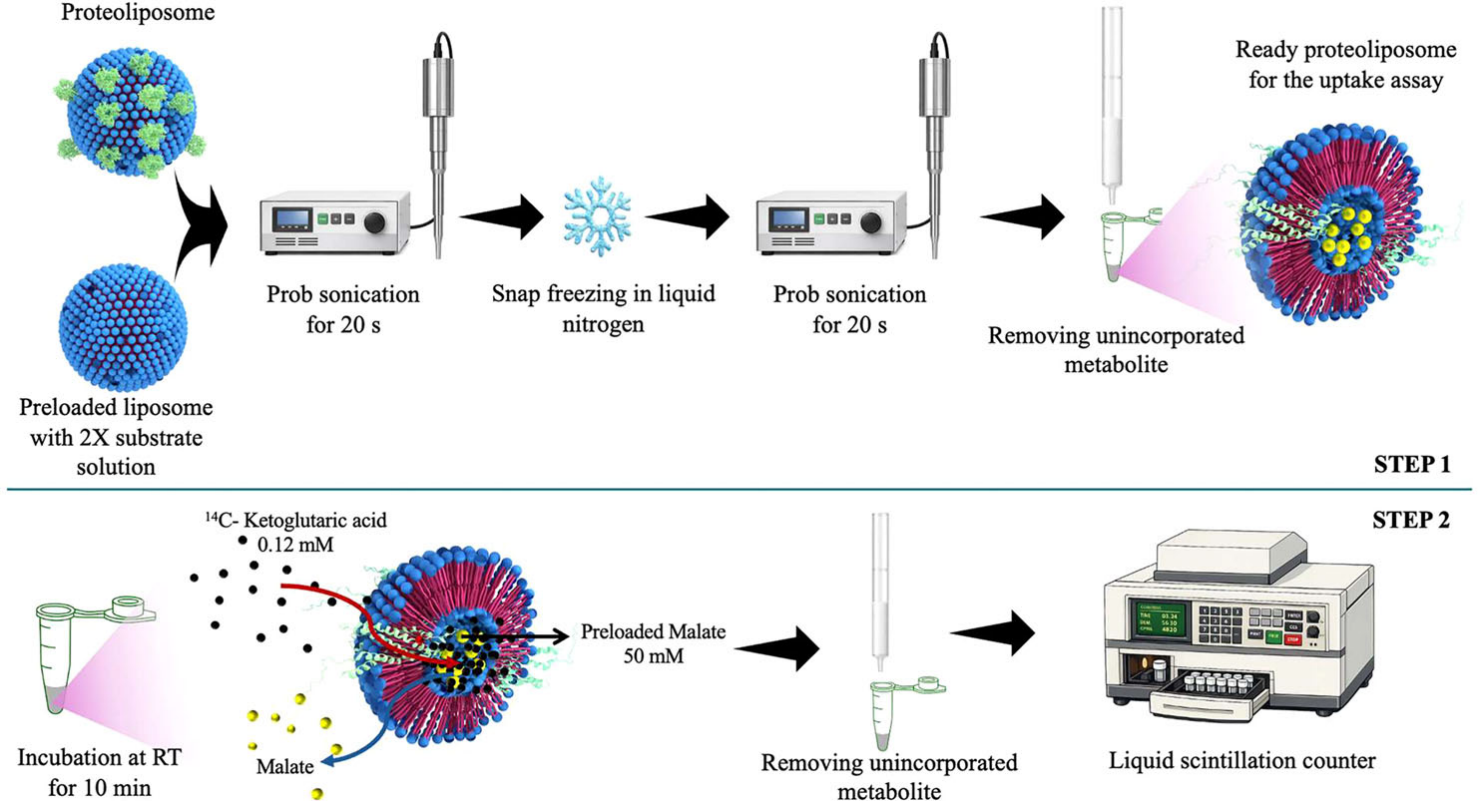
Schematic illustration of proteoliposome preparation and transport activity assay workflow. STEP 1: Liposomes and proteins are initially prepared separately, then reconstituted via probe sonication and snap‐freezing, yielding uniform proteoliposomes incorporating active transport proteins. STEP 2: The preloaded proteoliposome was then mixed with radiolabeled metabolites, and after a 10 min incubation at room temperature, unincorporated tracer was removed and radioactivity was measured using a liquid scintillation counter.

Proteins embedded with asolectin are further reconstituted into a substate‐preloaded liposome using probe sonication and a freeze‐thaw cycle to ensure uniform incorporation and membrane integrity. The proteoliposomes were prepared for the evaluation of transport activity using a radioactive tracer. Resultant proteoliposomes from Phase 3 were incubated with radioactive tracer at room temperature for 10 min. The radioactive tracer enables sensitive and qualitative measurement of proteoliposome uptake, providing an assessment of the functional incorporation of the protein of interest.

After incubation, the uninternalized radioactive tracer was removed by desalting with a size‐exclusion column (the column efficiency was evaluated as shown in the Supporting Information, 1.2. Column Efficiency, Fig. 1), and the proteoliposome fractions were quantified by measuring radioactivity with a scintillation counter. This approach enables a direct comparison of uptake efficiency between experimental proteoliposomes and controls. Phase 3 necessitates the following components:

(1) Proteoliposomes are reconstituted in Phase 2.
(2) A suitable radioactive tracer compatible with the protein or cargo of interest.
(3) Buffer and reagent for washing and equilibration.
(4) Detection equipment for radioactivity measurement (e.g., scintillation counter).

#### Experimental control

A representative positive control from the plant, proteoliposomes containing a previously validated AtDTC protein that is efficiently internalized, is used to ensure that the radioactive tracer and the uptake assay are functioning correctly. Negative background control includes liposomes without expressed protein, which allows assessment of background signal and non‐specific tracer uptake. To confirm that differences in uptake are not due to variations in proteoliposome concentration, total proteoliposome content is normalized to protein concentration (see Supporting Information, 1.3. Protein Quantification Using the BSA Assay), and enzymatic activity is monitored for all experimental samples.

### Limitations of the Protocols

Reconstituted proteoliposome‐based transport systems are predicted to reflect key steps underlying membrane insertion and functional activity of small‐molecule transporters (Amati et al., 2020). To evaluate transporter competence, these protocols assess whether proteins expressed via WG‐CFPS can be incorporated into liposomes and mediate tracer uptake within a reconstituted membrane environment. However, successful protein expression, membrane insertion, and transport activity in this in vitro system do not guarantee equivalent function in a native cellular or organellar context. Several additional biological and biophysical factors regulate transport activity in vitro and in vivo, which this method cannot address (Wang et al., 2024). These include native membrane lipid composition and asymmetry, membrane potential, post‐translational modifications, regulatory protein interactions, and physiological assembly states, all of which may influence transport efficiency. Although detergent‐assisted in vitro reconstitution and asolectin liposome enable efficient membrane insertion, they do not fully recapitulate the complexity of endogenous membranes and translation (Nozawa et al., 2007).

Rather, this method provides a framework to rank‐order transporter constructs based on solubility, membrane insertion competence, and in vitro transport activity. It should therefore be applied as a screening and validation tool within that context, with subsequent functional confirmation in more complex cellular systems required to establish physiological relevance. Furthermore, the tagged transporters require that the binding site face the extra‐liposomal space to generate a signal that changes transport activity. Therefore, we do not expect orientation heterogeneity to disproportionately bias these results, although we cannot entirely rule out the possibility that the treatment influences insertion orientation.

*CAUTION*: EDTA is toxic, so avoid exposure.

*CAUTION*: NaOH is corrosive and toxic.

*CAUTION*: RIPA causes serious eye irritation.

*CAUTION*: Radioactive reagents must be used in a designated radioactive room, and a safety shield and reagent‐impermeable protective gloves should be worn.

## Basic Protocol 1 PLASMID DESIGN AND DEVELOPMENT

In this phase, restriction enzymes are selected based on the vector map (e.g., EcoRV‐HF for pEU‐E01‐MCS). For successful Gibson assembly, annealing sequences for the forward and reverse strands are strategically selected at 25 and 27 nucleotides from the vector backbone, respectively. Furthermore, appropriate forward and reverse primers must be selected for colony PCR screening to verify successful insertion of the target sequence.

Scope of routine molecular biology procedures include: cloning, transformation, colony PCR, plasmid preparation, and sequence validation steps are standard molecular biology procedures. They are retained primarily to establish the workflow and to provide the critical parameters required for reproducibility. Kit‐dependent operations should be performed according to the current manufacturer's instructions, while the article emphasizes the experimental decisions that directly affect downstream membrane protein expression and functional reconstitution. In particular, correct insert identity and sequence integrity are treated as the release criteria for progression to cell‐free expression.

This protocol describes the design, assembly, and validation of a plasmid for WG‐CFPS using the pEU‐E01‐MCS expression vector. This includes plasmid preparation, vector linearization, DNA insert assembly, bacterial transformation, colony screening, and sequence validation. Upon successful completion, you will be ready for downstream cell‐free protein expression and proteoliposome preparation. When performed correctly, positive transformants produce the expected PCR amplicon size, and sequencing confirms accurate insertion of the target gene into the expression vector.

### Materials

*E. coli* containing pEU‐E01‐MCS vector
LB‐agar plates containing ampicillin (see recipe)
LB Lennox (Thermo Fisher Scientific, cat. no. BP1427‐500)
100 mg/ml ampicillin (Sigma‐Aldrich, cat. no. A5354‐10ML)
QIAGEN Plasmid Plus Midi kit (QIAGEN, cat. no. 12943)
H_2_O, nuclease‐free (Invitrogen, cat. no. AM9914G; Sigma‐Aldrich, cat. no. W4502‐1010X50ML)
10× rCutSmart buffer (New England Biolabs, cat. no. B6004S)
EcoRV‐HF (New England Biolabs, cat. no. R3195S)
Wizard HMW DNA extraction kit (Promega, cat. no. A9281)
Q5 reaction buffer pack, 5× (New England Biolabs, cat. no. B9027S)
Deoxynucleotide (dNTP) solution mix (New England Biolabs, cat. no. N0447S)
Q5 Hot Start High‐Fidelity DNA polymerase (New England Biolabs, cat. no. M0493S)
50× Tris‐acetate‐EDTA (TAE) buffer (Thermo Fisher Scientific, cat. no. B49)
Agarose, certified molecular biology (Bio‐Rad, cat. no. 1613102)
10,000× GelRed nucleic acid gel stain ( (Biotium, cat. no. 41003)
6× purple gel loading dye (New England Biolabs, cat. no. B7024S)
NEBuilder HiFi DNA assembly master mix (New England Biolabs, cat. no. M5520A)
E. cloni 10G SOLOs chemically competent cells (Biosearch Technologies, cat. no. 60106‐1)
40% glycerol

Incubator (e.g., Thermo Fisher Scientific, Heratherm compact microbiological incubator)
50‐ml Falcon tube
Shaker incubator (Thermo Fisher Scientific, MaxQ 4450 benchtop orbital shaker)
Ice bucket and ice
Thermocycler (Bio‐Rad, T100)
Vortex mixer (Scientific Industries, cat. no. SI‐A236)
Minispin centrifuge (USA Scientific, model IR; Azer Scientific, model D1008)
Benchtop microcentrifuge (Fisher Scientific, Sorvall Legend Micro 21R)
Milli‐Q water system (Millipore Sigma, Milli‐Q IQ 7000)
Gel electrophoresis system (Bio‐Rad, PowerPac HC high‐current power supply; Thermo Fisher Scientific, Owl EasyCast B1A gel electrophoresis system)
1.5‐ and 15‐ml tubes

#### Revival of E. coli containing pEU‐E01‐MCS vector

1 Streak *E. coli* containing the vector of interest (pEU‐E01‐MCS) onto an LB‐agar plate containing ampicillin.
2 Incubate overnight at 37°C.

#### Expansion of E. coli and plasmid extraction

3 Prepare 500 ml of 2% (w/v) autoclaved LB medium.
4 Add 25 ml of autoclaved LB medium (2%) and 25 µl of 100 mg/ml ampicillin (1000× stock) to a 50‐ml Falcon tube. Keep the tube at a 45° angle to improve aeration.
5 Inoculate with a single colony and incubate overnight at 37°C while shaking at 180 rpm.
6 Extract plasmid DNA using a Midi Prep kit and elute with nuclease‐free water.
7 Measure the plasmid DNA concentration.

#### Restriction enzyme digest (e.g., EcoRV‐HF)

8 Dilute plasmid DNA to 150 ng/µl in nuclease‐free water and prepare 100 µl total volume.
9 Thaw 10× rCutSmart buffer at room temperature, then place on ice.
10 Assemble the digestion reaction (100 µl total volume) on ice as follows:
  80 µl of 150 ng/µl plasmid
  10 µl of 10× rCutSmart buffer
  10 µl EcoRV‐HF.
11 Incubate at 37°C for 4 hr in a thermocycler.

#### Purify DNA

12 Purify the digested vector using the Wizard SV gel and PCR Clean‐Up system immediately after digestion to remove the enzyme and buffer, skipping gel extraction if desired.
13 Elute DNA in 40 µl nuclease‐free water.
14 Measure the digested and cleaned‐up DNA concentration.
  Pause point: A digested and cleaned‐up vector could be stored up to 2 weeks at −20°C.

#### Preparation of the DNA insert

If linear dsDNA is ordered commercially, resuspend it directly in nuclease‐free water with a concentration of 100 nM and proceed to assembly. Otherwise, follow steps 15 to 28 for amplification and purification. Annealing sequences for forward and reverse strands were selected at 25 and 27 nucleotides, respectively, from the pEU‐E01‐MCS vector.

##### PCR amplification of the insert from the vector

15 Prepare the PCR reaction × 2 on ice as follows:
  37 µl H_2_O, nuclease‐free
  10 µl of 5× Q5 reaction buffer
  1 µl of 10 mM dNTPs
  0.5 µl forward primer
  0.5 µl reverse primer
  0.5 µl of 20 ng/µl template
  0.5 µl Q5 Hot Start DNA polymerase
  50 µl total volume.
16 Vortex briefly and spin in a Minispin centrifuge.
17 Run PCR using the following program (extension rate of 30 s/kb):

**Table:** 

Action	Temperature (°C)	Time (s)
Initial denaturation	98	60
35 cycles of:	98	10
	60	30
	72	30
Final extension	72	120
Hold	4

###### DNA cleanup: Agarose gel purification

18 Prepare 1× TAE buffer by diluting 8 ml 50× TAE in 392 ml Milli‐Q water.
19 Dissolve 750 mg agarose in 75 ml 1× TAE by microwave heating.
20 Cast gel with 5‐well gel comb and allow to solidify for 1 hr at room temperature.
21 Mix the same volume of the sample with the 2× GelRed/purple dye mixture.
22 Load all 200 µl to the desired wells and add ladder to the desired well.
23 Run gel at 120 V for 45 min.
24 Image gel using a GelRed detection setting.
25 Cut the desired DNA band under the UV light.
26 Extract and purify the PCR product using the Wizard SV Gel and PCR Clean‐Up system.
27 Elute the DNA with 30 µl nuclease‐free water.
28 Measure the cleaned‐up DNA concentration.

#### Construction of a cell‐free protein synthesis plasmid

29 Calculate the digested and cleaned‐up vector and insert the molar concentrations:
30 Prepare 8 µl of a vector:insert molar ratio of 1:2 (recommended: 50 nM vector, 100 nM insert).
31 For the negative control, add the same amount of nuclease‐free water instead of the gene of interest to the digested and cleaned‐up vector.
32 Mix 5 µl DNA mixture or negative control with 5 µl of 2× NEBuilder HIFI DNA assembly master mix.
33 Incubate in a thermocycler at 50°C for 20 min.
34 Cool at room temperature and keep on ice.

#### Transformation and colony screening

35 Chill two 1.5‐ml and two 15‐ml tubes on ice.
36 Thaw E. cloni 10G cells in ice for 20 min.
37 Divide cells into two 25‐µl tubes and transfer to two ice‐cold 1.5‐ml tubes.
38 Add 2 to 5 µl assembly reaction or negative control to 25 µl competent cells.
  Do not mix by pipetting up and down; mix with stirring using the tip.
39 Transfer the cells containing DNA to prechilled 15‐ml tubes and incubate on ice for 30 min.
40 Heat shock at 42°C for 45 s and return to ice for 2 min.
41 Add 1 ml of 2% LB medium and recover for 1 hr at 37°C while shaking at 200 rpm.
42 Plate serial dilutions of recovered cells on LB‐agar with a final concentration of 100 µg/ml ampicillin.
43 Incubate overnight at 37°C.

#### Colony PCR

Choose appropriate forward and reverse primers first (here, we chose vector‐specific primers as the sequence is reported in the supplementary).

44 Select 7 samples and 1 negative control colony and number them on the back of the plate.
  Avoid taking agarose from your plate; this can be a major inhibitor of your PCR reaction.
45 Take half of each colony and suspend in 5 µl nuclease‐free water.
  If the colonies are not big enough to take half of them, incubate plates overnight at room temperature.
46 Boil 5 to 10 minutes at 95°C in a thermocycler and using a benchtop microcentrifuge, centrifuge ~3 s at maximum speed, room temperature.
47 Set up the colony PCR mixture as follows:
  112 µl H_2_O, nuclease‐free
  30 µl of 5× Q5 reaction buffer
  3 µl of 10 mM dNTPs
  1.75 µl forward primer XS085
  1.75 µl reverse primer XS086
  1.5 µl Q5 Hot Start DNA polymerase
  150 µl total volume.
48 Vortex briefly and centrifuge ~3 s at maximum speed, room temperature.
49 Make 8 aliquots of 18.5 µl from the PCR product.
50 Add 3 µl of supernatant from the boiled colony to the aliquots’ PCR mixture.
51 Vortex and centrifuge ~3 s at maximum speed, room temperature.
52 Set up the thermocycler program as follows:

**Table:** 

Action	Temperature (°C)	Time (s)
Initial denaturation	98	60
35 cycles of:	98	10
	60	30
	72	30
Final Extension	72	120
Hold	4
53 Run colony PCR products on a 1% agarose gel to check purity and yield as described above in “DNA cleanup: Agarose gel purification.”
54 Choose the best positive colony (if insertion was successful, a band appears in a higher molecular weight).
55 Expand another half of colonies in 25 ml LB broth containing 100 µg/ml ampicillin.
56 Make the frozen stock from each inoculate after mixing 1:1 cell and 40% glycerol and store at –80°C.
57 Extract plasmid DNA using a Midi Prep kit and elute with nuclease‐free water.
58 Measure the plasmid DNA concentration.
59 The positive extracted plasmids were sent for whole plasmid sequencing.
60 After sequence alignment, use positive stock to expand the clone in 100 ml of 2% LB broth containing 100 µg/ml ampicillin overnight at 37°C.
  Keep the Falcon tube at a 45° angle to improve aeration.
61 Extract the plasmid using the Midi Prep kit.
62 Adjust the concentration of plasmid to 500 ng/µl.
  Pause point: Plasmids can be stored at –20°C for up to 3 months.

## Basic Protocol 2 CELL‐FREE PROTEIN EXPRESSION

To enhance the solubility of the mitochondrial carriers and promote their proper folding, the target proteins are translated in the presence of Brij‐35 and asolectin liposomes (Nozawa et al., 2007). During the purification step, the solution is gently mixed by pipetting to ensure all proteoliposomes attached to the dialysis membrane are recovered. Simple centrifugation and washing steps then separate the successfully incorporated proteoliposomes from unincorporated proteins.

Critical methodology includes protein incorporation and membrane‐competence screening. During bilayer‐dialysis expression, the 500‐µl translation mixture is layered beneath the feeding buffer and incubated at 15°C for 72 hr. Following the expression, gentle recovery of material associated with the dialysis membrane is followed by centrifugation for 20 min at 20,000 × *g*, 4°C, and three washing cycles. This workflow enriches proteoliposomes while removing soluble wheat‐germ components. Importantly, pellet formation or co‐migration alone is considered an incorporation screen, not proof of functional reconstitution. A protein‐free liposome control is processed in parallel to estimate nonspecific co‐migration, and protein concentration is normalized using the BSA assay. Functional incorporation is subsequently established in Basic Protocol 3 by substrate uptake, thereby separating membrane association from transport competence. Critical methodology: preparation of asolectin liposomes.

Liposome quality is important because the lipid phase is present during translation and becomes the membrane environment in which the transporter is incorporated. Asolectin is first dispersed in feeding buffer, incubated, vortexed, probe‐sonicated on ice, and extruded through a 200‐nm membrane. Probe sonication is performed in repeated short cycles with cooling to minimize heating and foaming. The manuscript states that bath sonication is ineffective under these conditions. After extrusion, centrifugation for 5 min at 5000 × *g*, room temperature, removes visible debris before the liposomes are introduced into the translation mixture. These parameters should be maintained consistently between experimental and background control preparations because differences in lipid dispersion can alter apparent protein incorporation and particle recovery (critical methodology: liposome preparation and detergent selection). The membrane‐protein expression step is the central transition from a conventional cell‐free translation reaction to a membrane‐competent proteoliposome. In this protocol, asolectin provides the lipid matrix, and Brij‐35 is incorporated into the translation mixture at a final concentration of 0.04%. The detergent is used as a mild, non‐ionic solubilizing environment during synthesis so that hydrophobic transmembrane regions remain soluble while the nascent protein can associate with the lipid bilayer. The protocol, therefore, avoids a separate post‐expression detergent extraction and removal step. Because the present study did not perform a head‐to‐head detergent screen, Brij‐35 should be regarded as the selected condition used here rather than as a universally optimal detergent. When adapting the workflow to another transporter, detergent concentration should be treated as an empirical optimization variable, with membrane incorporation, aggregation, and downstream transport activity used as the decision criteria rather than protein yield alone.

This section describes the synthesis of membrane proteins in a WG‐CFPS and their co‐translational incorporation into azolectin liposome to generate a proteoliposome. The procedure includes in vitro transcription, detergent‐assisted translation, bilayer dialysis‐based protein expression, and proteoliposome purification. Successful execution yields a purified functionally reconstituted proteoliposome suitable for downstream biochemical and transport activity assays. When performed correctly, the resulting proteoliposomes contain the target membrane protein embedded within lipid bilayers with minimal aggregation and background contamination.

### Materials

Plasmid DNA (see Basic Protocol 1)
Proteoliposome BD expression kit bilayer‐dialysis reaction format (CellFree Science, cat. no. CFS‐CPLE‐BD)
H_2_O, nuclease‐free (Invitrogen, cat. no. AM9914G; Sigma‐Aldrich, cat. no. W4502‐1010X50ML)
Brij 35 detergent, 30% aqueous solution (Millipore Sigma, cat. no. 203724‐100ML)
PIPES (Millipore Sigma, cat. no. P6757‐100G)
HEPES (Millipore Sigma, cat. no. H3375‐250G)
NaOH (Millipore Sigma, cat. no. S5881‐500G)

Ice bucket and ice
Vortex mixer (Scientific Industries, cat. no. SI‐A236)
Benchtop microcentrifuge (Fisher Scientific, Sorvall Legend Micro 21R)
Thermocycler (Bio‐Rad, T100)
1.5‐, 15‐, and 50‐ml tubes
Probe ultrasonic system (Banson, 450 Digital Sonifier)
Whatman Nuclepore polycarbonate track‐etched membranes filters (Cytiva, cat. no. 800309)
Slide‐A‐Lyzer MINI dialysis device, 10 kDa MWCO, 50‐ml conical tube (Thermo Fisher Scientific, cat. no. 88404)

#### In vitro transcription using plasmid DNA template

1 Thaw the plasmid DNA (circular, 500 ng/µl) at room temperature.
2 Remove aliquoted transcription buffer LM (5×), NTP mix, RNase inhibitor, and SP6 RNA polymerase (from the Proteoliposome BD expression kit) from −80°C, thaw at room temperature, and keep them on ice.
  Always keep the thawed enzyme and reagents on ice to prevent loss of activity.
3 After thawing, briefly vortex and centrifuge all reagents ~3 s at maximum speed, room temperature.
4 Assemble the transcription reaction for each sample or negative control (150 µl volume) on ice as follows:
  71.31 µl H_2_O, nuclease‐free
  30 µl of 5× transcription buffer LM (1× final)
  15 µl of 25 mM NTP mix (2.5 mM final)
  1.87 µl of 80 U/µl RNase inhibitor (1 U/µl final)
  1.87 µl of 80 U/µl SP6 RNA polymerase (1 U/µl final)
  30 µl of 500 ng/µl circular plasmid DNA (100 ng/µl final).
  For negative control, instead of plasmid, add nuclease‐free water.
  Do not use DEPC‐treated water; use commercially prepared nuclease‐free water for all steps.
5 Gently mix the mixture by pipetting and incubate at 37°C for 6 hr in a thermocycler.
6 After incubation, leave the reaction tube at room temperature for 10 to 15 min.
  Do not place the reaction tube on ice.
7 If white precipitate forms, centrifuge 5 min at 10,000 × *g*, 4°C, and use the supernatant.

#### Preparation of Brij‐35‐containing feeding buffer

8 Prepare 1 ml of 4.8% Brij‐35 by adding 160 µl of 30% Brij‐35 to 840 µl nuclease‐free water.
9 Prepare 48 ml of 1× SUB‐AMIX SGC feeding buffer as follows, adding components (from the Proteoliposome BD expression kit) in the order listed and mixing after each addition to prevent precipitation.
  42.8 ml H_2_O, nuclease‐free
  0.4 ml of 4.8% Brij‐35 (0.04% final)
  1.2 ml of 40× SUB‐AMIX SGC (S1) (1× final)
  1.2 ml of 40× SUB‐AMIX SGC (S2) (1× final)
  1.2 ml of 40× SUB‐AMIX SGC (S3) (1× final)
  1.2 ml of 40× SUB‐AMIX SGC (S4) (1× final).
10 Keep the feeding buffer on ice.

#### Preparation of uniflagellar asolectin liposome

11 Slowly add 400 µl of 1× SUB‐AMIX SGC feeding buffer to the bottom center of an asolectin vial (from the Proteoliposome BD expression kit) at room temperature.
12 Close the vial and incubate at room temperature for 10 min.
13 Vortex for 1 min to disperse the lipids.
14 Transfer the suspension to a pre‐chilled 15‐ml tube and place it in watery ice.
15 Probe‐sonicate for 30 s, rest on ice for 2 min, and repeat 3 times until a clear yellowish solution is observed.
  Avoid making bubbles or foam during probe‐sonication in all steps.
  Bath sonication is not effective.
16 Extrude the suspension through a 200‐nm membrane 10 times.
17 Centrifuge the extruded liposome 5 min at 5000 × *g*, room temperature, to remove debris, then collect the supernatant.
18 To prepare liposomes for background, follow the steps in “Asolectin liposome for negative background control” below.

#### Asolectin liposome for negative background control

19 Slowly add 400 µl of 1× SUB‐AMIX SGC feeding buffer to the bottom center of an asolectin vial.
20 Close the vial and incubate at room temperature for 10 min.
21 Vortex for 1 min to disperse the lipids.
  Do not sonicate or centrifuge and directly use the liposome for background control preparation for assembly of wheat germ translation mixture.

#### Assembly of what germ translation mixture

Follow the exact same procedure for making the negative background liposome.

22 Thaw the aliquoted WEPRO and creatine kinase (from the Proteoliposome BD expression kit) on ice 20 to 30 min before use.
23 Prepare 1 ml of 5.1% Brij‐35 by diluting 170 µl of 30% Brij‐35 with 830 µl nuclease‐free water.
  Use the negative control of the transcription reaction as the mRNA solution for the translation mixture for making the negative background control liposome.
24 Assemble the translation mixture (500 µl total) as follows:
  125 µl mRNA (1/4 volume final)
  47 µl of 1× SUB‐AMIX SGC
  2 µl of 5.1% Brij‐35 (0.04% final)
  125 µl of 240 OD WEPRO7240 (60 OD final)
  1 µl of 20 mg/ml creatine kinase (40 µg/ml final)
  200 µl of 25 mg/ml asolectin liposome (10 mg/ml final)

#### Bilayer dialysis (BD) translation reaction

Follow the exact same procedure for making the negative background liposome.

25 Place 43 ml of feeding buffer into a 50‐ml tube.
26 Rinse a Slide‐A‐Lyzer MINI dialysis device (10 kDa MWCO) with 4 ml nuclease‐free water to confirm integrity.
27 Discard the 4 ml nuclease‐free water.
28 Insert the dialysis cup into the feeding buffer immediately and add 2 ml of feeding buffer to the cup.
  Do not dry the membrane of the Slide‐A‐Lyzer MINI dialysis device.
29 Carefully and gently layer 500 µl of prepared translation mixture beneath the buffer by slowly dispensing at the bottom of the cup.
30 Close the cap without shaking and incubate at 15°C for 72 hr.

#### Purification of proteoliposomes

31 Discard the 43 ml of 1× SUB‐AMIX SGC feeding buffer from the 50‐ml tube.
32 Gently mix the 2.5 ml solution inside the cup by pipetting up and down.
33 Split the 2.5 ml solution into three 1.5‐ml tubes.
34 Centrifuge 20 min at 20,000 × *g*, 4°C.
35 Remove 90% of the supernatant and resuspend the pellet in the buffer of interest (here we used 25 mM HEPES‐NaOH, pH 7.4 for SLC25A11 and 10 mM PIPES‐NaOH, pH 6.5 for SLC25A21, AtDTC, and negative background control liposome).
36 Pool the pellets and resuspend them in 1 ml buffer.
37 Repeat centrifugation as in step 34 and wash two additional times.
38 Resuspend pellet in 500 µl buffer and disperse by pipetting.
  If aggregation persists, probe‐sonication for 20 s on ice.
39 Measure the protein concentration using the BSA assay. See Supporting Information Supplementary Materials, 1.3. Protein Quantification Using the BSA Assay.
  It is recommended to use the proteoliposomes fresh, as they were used in this protocol.
  Pause point: Resuspended pellet can be snap‐frozen in liquid nitrogen and stored for 2 days.

## Basic Protocol 3 ACTIVE RECONSTITUTION AND VALIDATION OF TRANSPORT ACTIVITY

Proteins embedded in asolectin are reconstituted into substrate‐preloaded liposomes using probe sonication and freeze‐thaw cycles to ensure uniform membrane integrity. Buffer selection is critical; for instance, because malate interacts with the PIPES‐NaOH buffer used when evaluating SLC25A11, PIPES is excluded and replaced with HEPES‐NaOH (pH 7.4). Following active reconstitution, size‐exclusion chromatography is used to remove uninternalized radioactive tracer and unincorporated substrates, thereby enabling accurate quantification of transport‐mediated uptake.

Critical controls and interpretation. Three control levels are recommended within the workflow: (i) a protein‐free liposome control to quantify nonspecific tracer association; (ii) a validated positive transporter control, e.g., AtDTC, to confirm that the reconstitution and tracer‐assay system is functional; and (iii) a competition condition containing excess unlabeled substrate to assess substrate‐dependent uptake. Protein concentration and proteoliposome preparation should be normalized across samples before comparing activities. These controls distinguish failures of protein expression or membrane incorporation from failures of the transport assay itself, thereby reducing the risk of interpreting a negative uptake result as evidence of transporter inactivity. Critical methodology: Removal of external substrate and transport‐assay design. After active reconstitution, size‐exclusion chromatography is used to remove unincorporated substrate before the uptake measurement. The column is equilibrated with 9 ml of the appropriate buffer, 150 µl of proteoliposome suspension is loaded, the specified wash volume is discarded, and the 500‐µl eluate is collected. This separation is essential because residual external substrate would increase the measured radioactive signal independently of transport. For the transport assay, proteoliposomes and negative background liposomes are pre‐equilibrated for 5 min, combined with radiolabeled substrate, and incubated for 10 min at room temperature, as reported. Competition experiments include a cold substrate as an additional specificity control. Samples are processed identically through the column before scintillation counting. Transport activity is therefore interpreted relative to the protein‐free background and, where applicable, competition controls rather than from absolute radioactivity alone. Critical methodology: buffer selection and substrate compatibility. Buffer composition is not interchangeable across transporter assays. For SLC25A11, malate interacts with PIPES under the conditions tested in this workflow; therefore, HEPES‐NaOH at pH 7.4 is used for the malate‐containing substrate and equilibration solutions. SLC25A21, AtDTC, and the corresponding background controls use PIPES‐NaOH at pH 6.5 as specified in the procedure. These buffer choices are part of the functional assay design and should be retained when reproducing the reported experiments. When adapting the assay to a different substrate or transporter, buffer compatibility should be evaluated before interpreting uptake differences as transporter activity. Critical methodology: active reconstitution and membrane integrity. The purpose of the second reconstitution step is to transfer the transporter‐containing asolectin material into a substrate‐preloaded lipid population while preserving a sealed membrane compartment. The protocol combines probe sonication with a liquid‐nitrogen freeze/thaw cycle: the protein–asolectin suspension is mixed 1:1 with 80 mg/ml substrate‐containing asolectin, probe‐sonicated, flash‐frozen for 30 s, thawed at room temperature, cooled on ice, and probe‐sonicated again. These operations are performed under controlled cooling because excessive heating or foaming can alter membrane integrity and reproducibility. The resulting proteoliposomes are kept on ice until the assay.

This section of the protocol describes the active reconstitution of cell‐free‐expressed membrane protein into substrate‐preloaded liposomes and the measurement of their transport activity using radiolabeled substrate. The procedure involves proteoliposome activation, removal of external substrates by size‐exclusion chromatography, transport assays under defined buffer conditions, and quantification of substrate uptake by liquid scintillation counting. Successful completion of the protocol yields functional proteoliposomes suitable for evaluating transport‐mediated substrate exchange, substrate specificity, and competitive inhibition. When performed correctly, transport active proteoliposomes exhibit significantly higher radiolabeled substrate uptake than negative background control liposomes.

### Materials

PIPES (Millipore Sigma, cat. no. P6757‐100G)
NaOH (Millipore Sigma, cat. no. S5881‐500G)
HEPES (Millipore Sigma, cat. no. H3375‐250G)
Potassium d‐gluconate (Millipore Sigma, cat. no. G4500‐100G)
l‐(‐)‐Malic acid (Millipore Sigma, cat. no. 112577‐25G)
α‐Ketoglutaric acid disodium salt dihydrate (Millipore Sigma, cat. no. 75892‐25G)
d‐Gluconic acid sodium salt (Millipore Sigma, cat. no. G9005‐500G)
Sodium acetate, anhydrous (Millipore Sigma, cat. no. W302406)
Asolectin (see Basic Protocol 2)
H_2_O, nuclease‐free (Invitrogen, cat. no. AM9914G; Sigma‐Aldrich, cat. no. W4502‐1010X50ML)
Ketoglutaric acid, a‐[1‐14C] sodium salt (American Radiolabeled chemicals, cat. no. ARC0319‐50µCi)
EcoLite liquid scintillation cocktail (MP Biomedicals, cat. no. 01882475‐CF)

pH meter (Fisher Scientific, Accumet AB315 benchtop laboratory pH/mV meter)
Nalgene Rapid‐Flow sterile filter, 0.2‐µm (Thermo Fisher Scientific, cat. no. 595‐3320)
Vortex mixer (Scientific Industries, cat. no. SI‐A236)
Probe ultrasonic system (Banson, 450 Digital Sonifier)
Ice bucket and ice
15‐ml tubes
Flash freeze container with liquid nitrogen
qEVsingle, 70‐nm column (IZON, 70 nm series)
1.5‐ml Eppendorf tubes
20‐ml scintillation glass vial (Duran Wheaton Kimble, cat. no. 986560)
Liquid scintillation counter (Beckman Coulter LS6500)

Follow the exact same procedure for making the negative background liposome.

1 Prepare 500 ml of 60 mM PIPES‐NaOH pH 6.5 buffer solution.
2 Prepare 500 ml of 100 mM HEPES‐NaOH pH 7.4 buffer solution.
3 Filter the prepared buffer solutions using a 0.2‐µm filter.
4 Prepare 10 ml of 2× substrate solution: 100 mM HEPES‐NaOH pH 7.4, 15 mM potassium gluconate, 50 mM malate (for SLC25A11).
5 Prepare 10 ml of 2× substrate solution: 60 mM PIPES‐NaOH pH 6.5, 15 mM potassium gluconate, 50 mM α‐ketoglutaric acid (for SLC25A21, background control liposomes, and AtDTC).
6 Prepare equilibration buffer: 10 mM PIPES‐NaOH pH 6.5, 40 mM potassium gluconate, 100 mM sodium gluconate (for SLC25A21, background control liposomes, and AtDTC).
7 Prepare equilibration buffer: 25 mM HEPES‐NaOH pH 6.5, 40 mM potassium gluconate, 100 mM sodium gluconate (for SLC25A11).
8 Filter the prepared equilibration buffer solution using a 0.2‐µm filter.
  Due to the interaction of malate with the PIPES‐NaOH buffer for evaluating SLC25A11, PIPES was excluded from the assay system. Instead of 60 mM PIPES‐NaOH pH 6.5, and 10 mM PIPES‐NaOH pH 6.5, 100 mM HEPES‐NaOH pH 7.4, and 25 mM HEPES‐NaOH pH 7.4 were used to prepare the 2× substrate solution with malate and the equilibration solution, respectively.
9 Prepare elution solution: 200 mM sodium acetate.
10 Filter the prepared elution solution using a 0.2‐µm filter.

#### Preparation of a substrate preloaded liposome

11 Prepare at least 300 µl of 80 mg/ml of asolectin using the 2× substrate solution for each sample.
12 Slowly add buffer to the bottom center of an asolectin vial, wait for 10 min, vortex for 1 min, and then pool them.
13 Probe‐sonicate the suspension (10% amplitude) on ice until the suspension becomes transparent.
14 If using a frozen protein‐asolectin suspension, it is important to probe‐sonicate for 20 s on ice after thawing at room temperature.
15 Mix the probe‐sonicated protein‐asolectin suspension or background liposome with 80 mg/ml asolectin prepared using 2× substrate solution in a ratio of 1:1 (v/v).
16 Transfer the solution to a prechilled 15‐ml tube.
17 Probe‐sonicate the mixture for 20 s on ice.
18 Flash freeze the mixture in liquid nitrogen for 30 s and thaws at room temperature.
19 Chill the mixture on ice for 5 min.
20 Probe‐sonicate the mixture for 20 s on ice.
21 Keep the prepared active proteoliposome on ice until use.
22 Remove the upper cap, twist off the bottom cap, and discard the buffer from the Izon column.
23 The column efficiency was evaluated using EGFP proteoliposomes and a radioactive tracer. See Supporting Information, Supplementary Materials, 1.1. Column Efficiency.
24 Equilibrate the column with a total of 9 ml of the appropriate prepared equilibration buffer.
25 Load 150 µl of reconstituted proteoliposome into the equilibrated column.
26 Add 750 µl of equilibrium buffer and discard filtrate.
27 Add 500 µl of equilibrium buffer and collect the elution.
  A total volume of 500 µl is sufficient for one experiment in triplicate.
28 Keep proteoliposomes on ice until use.

#### Transport activity of proteoliposomes

29 Remove the upper cap, twist off the bottom cap of the new column, and discard the buffer.
30 Equilibrate column with a total of 9 ml of 200 mM sodium acetate.
31 Incubate the proteoliposome and the negative background liposome for 5 min before the transport assay at room temperature.
32 Add 10 µl radioactive substrate (ketoglutaric acid, a‐[1‐14C]) and 10 µl nuclease‐free water to the 1.5‐ml Eppendorf tube.
33 For the competition assay, add 10 µl of radioactive substrate and 10 µl of cold substrate (here, cold α‐ketoglutaric acid at a final concentration of 10 mM).
34 Gently pipette up and down once to homogenize the proteoliposome or the negative background liposome.
35 Add 130 µl of the prepared proteoliposome or the negative background liposome to the corresponding substrate‐containing tube, then mix very gently by tapping the tube 3 times with a finger.
  Do not pipette up and down or vortex. Just mix gently by tapping three times with a finger.
36 Incubate at room temperature for the specified time (here, 10 min).
37 Load 150 µl of samples into the equilibrated column with a total of 9 ml of 200 mM sodium acetate.
38 Add 750 µl of sodium acetate and discard the filtrate.
39 Use a new tube to collect fractions.
40 Add 500 µl of 200 mM sodium acetate, then collect the eluate.
41 Add 10 ml of EcoLite scintillation liquid to the 20‐ml scintillation glass vials.
42 Add the whole elution fraction of the sample to the 10 ml of EcoLite scintillation liquid.
43 Vortex for 5 to 10 s.
44 Measure the radioactivity using a liquid scintillation counter.
45 Calculate the activity.
  See Supporting Information, Supplementary Materials, 1.4. Calculation of the Proteoliposome Substrate Rate of Uptake (Activity).

## REAGENTS AND SOLUTIONS

### LB‐agar plates containing ampicillin

Add 10 g agar (Sigma‐Aldrich, cat. no. 05039‐500G) and 10 g LB Lennox (Thermo Fisher Scientific, cat. no. BP1427‐500) to a 1‐L Erlenmeyer flask. Add 500 ml milli‐Q water and shake gently for 1 min. Cover the flask opening with aluminum foil and autoclave. At this stage, the solution must be clear and yellowish. Allow the solution to cool on the bench for several minutes until warm enough to add the antibiotic (do not cool below 55°C; the solution should be hot but not too hot to cause burns). Add 500 µl ampicillin (100 mg/ml, 1000× stock; Sigma‐Aldrich, cat. no. A5354‐10ML) to 500 ml medium to obtain a final concentration of 100 µg/ml. Turn on a flame and dispense 24 ml medium into each plate. Replace lids, turn off flame, and allow plates to solidify overnight at room temperature. Store plates up to 2 weeks at 4°C until use.

## COMMENTARY

### Critical Parameters

#### Confirm sequence integrity before expression

Correct insert identity and sequence integrity are the critical release criteria that must be met before progressing to cell‐free protein expression.

#### Design precise annealing sequences

Annealing sequences for the forward and reverse strands must be selected exactly at 25 and 27 nucleotides from the vector backbone to ensure successful Gibson assembly.

#### Prevent PCR inhibition during screening

When sampling colonies for PCR, entirely avoid taking agarose from the plate, as it acts as a major inhibitor to the reaction. If bacterial colonies are not large enough to easily harvest half of the colony, incubate the plates overnight at room temperature before sampling.

#### Control liposome sonication conditions

Prepare asolectin liposomes using probe sonication in repeated short cycles on ice to minimize heating and foaming; bath sonication is ineffective for this procedure.

#### Maintain reagent integrity

Keep enzymes and transcription reagents strictly on ice to prevent loss of activity and use commercially prepared nuclease‐free water rather than DEPC‐treated water for all steps. Following the transcription incubation step, leave the reaction tube at room temperature for 10 to 15 min and do not place it on ice.

#### Handle bilayer dialysis assembly carefully

Ensure the dialysis membrane does not dry out and carefully layer the 500 µl translation mixture beneath the feeding buffer by slowly dispensing it directly at the bottom of the dialysis cup.

#### Select target‐compatible buffer systems

Buffer composition is extremely sensitive and not interchangeable due to substrate interactions; e.g., use 100 mM HEPES‐NaOH (pH 7.4) for SLC25A11 to avoid malate interactions with PIPES, and 60 mM PIPES‐NaOH (pH 6.5) for SLC25A21 and AtDTC.

#### Regulate temperature during active reconstitution

Perform active reconstitution utilizing probe sonication and liquid‐nitrogen freeze‐thaw cycles under strict cooling controls to prevent excessive heating or foaming that alters membrane integrity.

#### Remove unincorporated external substrates

Entirely remove external substrates via size‐exclusion chromatography before measuring uptake, as residual external substrate will artificially inflate the measured radioactive signal.

#### Mix transport assays gently

During the transport assay setup, mix the proteoliposome and radiolabeled substrate very gently by tapping the tube three times with a finger; pipetting up and down or vortexing is strongly prohibited.

#### Normalize data for accurate comparison

Properly normalize the total proteoliposome content to the protein concentration prior to comparing activities between experimental samples and controls to obtain robust and reproducible results.

### Troubleshooting

Table 1 lists problems that may arise with this procedure along with their possible causes and solutions.

**Table 1:** Troubleshooting Guide for Proteoliposome Preparation and Functional Reconstitution of Human Mitochondrial Transporters Via Wheat Germ Cell‐Free Expression System

Problem	Possible cause	Solution
Colony PCR reaction is inhibited, yielding poor or no target amplification	Agarose was transferred from the plate during colony selection, which acts as a major PCR inhibitor; alternatively, the colonies may be too small for adequate sampling	Avoid taking agarose from the plate when picking colonies; if colonies are not big enough to sample, incubate the plates overnight at room temperature before screening
A white precipitate forms in the reaction tube	Precipitation occurs following the 6 hr in vitro transcription incubation at 37°C	Do not place the reaction tube on ice; centrifuge the mixture for 5 min at 10,000 × *g*, 4°C, and proceed with the protocol using only the resulting supernatant
Proteoliposome aggregation persists during the purification steps	The pelleted proteoliposomes fail to disperse completely after being resuspended in buffer and mixed by pipetting	Disperse the pellet by pipetting first; if aggregation persists, perform probe‐sonication on the sample for 20 s on ice
Bubbles or foam appear during liposome dispersion and active reconstitution	Improper sonication technique or the use of ineffective bath sonication	Perform probe‐sonication in repeated 30 s cycles on watery ice with 2 min resting periods; bath sonication is ineffective and must not be used
Standard assay buffer chemically interacts with the evaluation substrate, altering activity	Specific substrates directly interact with the assay buffer, e.g., the interaction between malate and PIPES‐NaOH	Exclude the interacting buffer from the assay system; e.g., replace PIPES‐NaOH with HEPES‐NaOH (pH 7.4) when evaluating SLC25A11 with malate
Radioactive measurements show an erroneously high signal independent of true transport	Residual external radiolabeled substrate remains in the suspension due to inadequate removal prior to the assay	Ensure rigorous size‐exclusion chromatography by equilibrating the column with 9 ml buffer, loading 150 µl suspension, and exclusively collecting the 500 µl eluate

### Understanding Results

The protocols described above enable the synthesis of human mitochondrial carrier protein using the WG‐CFPS system, followed by its incorporation into liposomes and functional evaluation using transport competition assays. To evaluate the method's performance in Phase 1, three samples (SLC25A11, SLC25A21, and AtDTC) were analyzed using colony PCR and whole‐plasmid sequencing, followed by sequence alignment of the DNA assembly. The results indicated 100% alignment between assembly and sequencing. Moving forward to Phase 2, the observations showed pellet formation after centrifugation. The protein concentration of each sample was determined using a BSA assay, as described in section 1.3 Protein Quantification Using the BSA Assay of the Supplementary Materials (see Supporting Information) and Coomassie‐stained SDS‐PAGE was performed to evaluate the expression and apparent molecular mass of AtDTC, SLC25A11, and SLC25A21 as shown in Supplementary Materials Figure 2 (see Supporting Information). The physicochemical properties of the proteoliposome were also evaluated, as shown in Supplementary Materials Figure 3 (see Supporting Information). The proteoliposome concentration did not differ significantly among the tested groups, indicating that the reconstitution process produced comparable particle numbers across the samples. However, a slight difference in particle size was observed. Proteoliposomes containing AtDTC showed a somewhat larger average diameter than those containing SLC2511 or SLC25A21. This difference may be related to the ultrasonication step during liposome preparation, which can influence vesicle size distribution and, occasionally, generate slightly larger vesicles depending on membrane composition and protein incorporation. Overall, these variations were minor and did not affect the overall reproducibility of the proteoliposome preparation. To evaluate the enzymatic activity of the SLC25A11, SLC25A21, and AtDTC as a representative control (Fig. 4), liposomes without protein incorporation were used as a background control, which was subtracted from the sample activity to remove the liposome effect on uptake.

**Figure 4:**
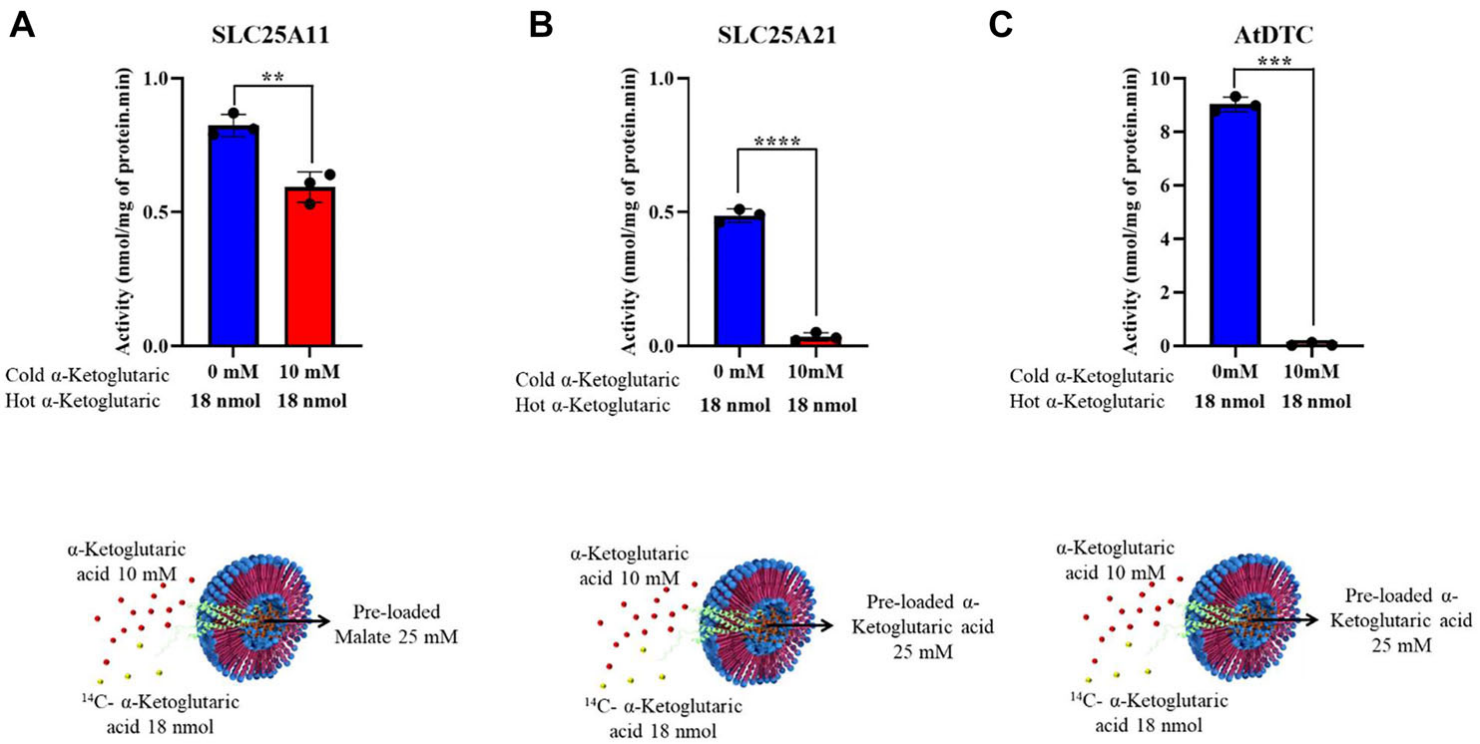
Functional characterization of reconstituted proteoliposome. Bar graph shows transport activity of (**A**) SLC25A11, (**B**) SLC25A21, and (**C**) AtDTC as a positive control. Schematic illustrations below each panel depict substrate translocation across the proteoliposome membrane through the protein, confirming directional transport and functional reconstitution. Data showed as mean; *n* = 3; *t*‐test significance indicated by ** is *p* < .005, *** is *p* < .0002, and **** is *p* < .0001.

The results indicated measurable transport activity of SLC25A11 proteoliposomes. In the standard assay, activity was ∼0.82 ± 0.04 nmol·mg of protein^−1^·min^−1^, which decreased to 0.6 ± 0.06 nmol·mg of protein^−1^·min^−1^ in the presence of 10 mM external α‐ketoglutarate. This reduction is consistent with the established ping‐pong transport mechanism of SLC25A11 (Cimadamore‐Werthein et al., 2024), in which the substrate interacts with a single binding site in a mutually exclusive manner. Increasing the external α‐ketoglutarate concentration accelerates α‐ketoglutarate binding and transport via SLC25A11, thereby reducing the difference between the standard and competition assays.

In contrast, similar modulation was not observed for SLC25A21. This carrier primarily transports α‐ketoglutaric acid across the inner mitochondrial membrane via a counter‐exchange mechanism (Fiermonte et al., 2001), exhibiting the same exchange behavior as AtDTC (Picault et al., 2002) under these assay conditions, confirming that the measured signals were derived from the incorporated transporters.

AtDTC showed the highest transport activity 9.03 ± 0.3 nmol·mg of protein^−1^·min^−1^, consistent with a previous comparison of its activity with that of other proteins (Picault et al., 2002). Although the wheat‐germ cell‐free system provides reliable protein synthesis and good reproducibility, the results suggest that it can be used to study human mitochondrial carriers, specifically those in the SLC25 family. WG‐CFPS reactions typically yield protein in the approximate range of 68 to 511 µg/ml reaction, although the actual yield varies depending on the membrane protein and its solubility. More soluble constructs generally show lower recovered protein concentrations (AtDTC), whereas poorly soluble membrane proteins like SLC25A21 may exhibit higher measurable remaining insoluble yields despite successful expression and lower transport activity.

### Time Considerations

Following plasmid construction for 5 days and cell‐free protein expression for 72 hr, uptake can be completed in 3 hr.

### Author Contributions

**Davood Kharaghani**: Formal analysis; investigation; methodology; writing—original draft; writing—review and editing. **Haotian Deng**: Validation. **Xiaoyang Su**: Methodology; supervision.

### Conflict of Interest

The authors declare no competing financial interests.

## Supporting information




*Supplementary materials: Proteoliposome preparation and functional reconstitution of human mitochondrial transporters via wheat germ cell‐free expression system*.

## Data Availability

The data that support the findings of this study are available from the corresponding author upon reasonable request.
