# Supplementary material for: Proteoliposome Preparation and Functional Reconstitution of Human Mitochondrial Transporters Via Wheat Germ Cell‐Free Expression System
Source: Curr Protoc. 2026 Oct 26;6(10):e70500. doi: 10.1002/cpz1.70500 (PMC13698637; doi:10.1002/cpz1.70500)
Supplement: Supplementary file 1 [file CPZ1-6-0-s001.docx]

**Supplementary Materials**

**Proteoliposome Preparation and Functional Reconstitution of Human Mitochondrial Transporters via Wheat Germ Cell-Free Expression System**

Davood Kharaghani*, Haotian Deng, Xiaoyang Su^*^

Department of Medicine, Rutgers-Robert Wood Johnson Medical School, Rutgers University, New Brunswick, NJ 08901, USA.

***Corresponding Author:** Xiaoyang Su, [xs137@rwjms.rutgers.edu](mailto:xs137@rwjms.rutgers.edu) and Davood Kharaghani, [kharaghani66@gmail.com](mailto:kharaghani66@gmail.com)

1.1. **Assembly and sequences**

**Supplementary Table.1**

| **Sample** | **Sequence** |
| --- | --- |
| **SLC25A11** | AACCACCTATCTACATCACCAAGATATGGCGGCGACGGCGAGTGCCGGGGCCGGCGGGATAGACGGGAAGCCCCGTACCTCCCCTAAGTCCGTCAAGTTCCTGTTTGGGGGCCTGGCCGGGATGGGAGCTACAGTTTTTGTCCAGCCCCTGGACCTGGTGAAGAACCGGATGCAGTTGAGCGGGGAAGGGGCCAAGACTCGAGAGTACAAAACCAGCTTCCATGCCCTCACCAGTATCCTGAAGGCAGAAGGCCTGAGGGGCATTTACACTGGGCTGTCGGCTGGCCTGCTGCGTCAGGCCACCTACACCACTACCCGCCTTGGCATCTATACCGTGCTGTTTGAGCGCCTGACTGGGGCTGATGGTACTCCCCCTGGCTTTCTGCTGAAGGCTGTGATTGGCATGACCGCAGGTGCCACTGGTGCCTTTGTGGGAACACCAGCCGAAGTGGCTCTTATCCGCATGACTGCCGATGGCCGGCTTCCAGCTGACCAGCGCCGTGGCTACAAAAATGTGTTTAACGCCCTGATTCGAATCACCCGGGAAGAGGGTGTCCTCACACTGTGGCGGGGCTGCATCCCTACCATGGCTCGGGCCGTCGTCGTCAATGCTGCCCAGCTCGCCTCCTACTCCCAATCCAAGCAGTTCTTACTGGACTCAGGCTACTTCTCTGACAACATCTTGTGCCACTTCTGTGCCAGCATGATCAGCGGTCTTGTCACCACTGCTGCCTCCATGCCTGTGGACATTGCCAAGACCCGAATCCAGAACATGCGGATGATTGATGGGAAGCCGGAATACAAGAACGGGCTGGACGTGCTGTTCAAAGTTGTCCGCTACGAGGGCTTCTTCAGCCTGTGGAAGGGCTTCACGCCGTACTATGCCCGCCTGGGCCCCCACACCGTCCTCACCTTCATCTTCTTGGAGCAGATGAACAAGGCCTACAAGCGTCTCTTCCTCAGTGGCTGAATCACTAGTTCTCGAGCTCGGTACCTG |
| **SLC25A21** | AACCACCTATCTACATCACCAAGATATGTCCGCCAAGCCTGAAGTCAGCTTAGTGCGCGAGGCTTCTCGGCAGATCGTGGCCGGTGGTTCTGCAGGTCTTGTAGAAATTTGCCTGATGCACCCCCTAGATGTGGTGAAAACCAGGTTTCAGATTCAGAGATGTGCAACCGATCCAAACAGTTATAAAAGCTTGGTAGACAGCTTTCGAATGATTTTCCAAATGGAAGGGTTATTTGGTTTTTACAAGGGAATTCTGCCACCTATCTTGGCTGAAACCCCAAAAAGAGCAGTGAAGTTTTTCACCTTTGAGCAGTACAAGAAATTGCTGGGATATGTGTCACTGTCACCAGCATTGACATTCGCCATTGCTGGATTGGGATCTGGACTAACAGAAGCCATTGTAGTTAACCCTTTTGAGGTAGTAAAAGTTGGCTTGCAAGCAAATCGGAACACATTTGCAGAGCAACCATCCACTGTGGGTTATGCAAGACAAATCATTAAGAAGGAAGGCTGGGGACTCCAGGGCCTCAACAAAGGATTAACTGCAACTTTGGGACGACATGGAGTTTTCAACATGGTTTATTTTGGCTTCTACTACAATGTCAAAAACATGATTCCTGTCAATAAGGATCCAATCTTGGAGTTTTGGAGAAAATTTGGGATTGGTCTTCTCTCGGGGACAATAGCCTCAGTCATTAACATCCCTTTTGATGTTGCCAAAAGTAGGATTCAAGGGCCTCAACCAGTTCCTGGAGAGATCAAGTACAGAACCTGTTTTAAAACAATGGCAACAGTCTATCAGGAAGAAGGGATTTTAGCTTTGTACAAAGGCCTGCTTCCCAAGATTATGAGACTTGGACCAGGTGGTGCAGTGATGCTGCTGGTTTATGAATACACCTATTCATGGCTTCAAGAGAACTGGTGAATCACTAGTTCTCGAGCTCGGTACCTG |
| **AtDTC** | AACCACCTATCTACATCACCAAGATATGGCGGAAGAGAAGAAAGCTCCAATCAGTGTCTGGACTACCGTGAAGCCCTTCGTCAATGGCGGTGCCTCTGGTATGCTCGCTACTTGCGTTATCCAGCCGATCGACATGATTAAGGTGAGGATTCAACTTGGTCAGGGATCTGCAGCCAGTATAACCACCAACATGCTTAAGAATGAGGGCGTTGGTGCCTTCTACAAGGGATTATCTGCTGGGTTGCTGAGGCAAGCAACTTACACGACAGCCCGTCTTGGATCATTCAAGTTGCTGACTGCAAAGGCAATTGAGTCTAATGATGGAAAGCCTCTACCGCTGTATCAGAAGGCACTATGTGGTCTGACAGCTGGTGCTATTGGTGCTTGCGTCGGTAGTCCAGCCGATCTAGCACTTATCAGAATGCAGGCCGATAATACTTTGCCGCTAGCTCAGCGCAGGAATTATACCAATGCTTTCCATGCGCTTACCCGTATTAGCGCTGATGAGGGAGTTTTAGCACTTTGGAAAGGGTGTGGGCCCACTGTGGTCAGAGCTATGGCTTTGAACATGGGAATGCTTGCATCTTATGATCAAAGTGCTGAATACATGAGAGATAATCTTGGTTTCGGGGAGATGTCTACGGTCGTAGGAGCAAGTGCTGTTTCTGGGTTCTGCGCTGCGGCTTGCAGTCTGCCATTTGACTTTGTCAAAACTCAGATTCAGAAAATGCAACCGGATGCTCAAGGAAAGTATCCATACACAGGTTCGCTCGATTGTGCGATGAAAACCTTAAAAGAAGGAGGACCTCTGAAATTTTACTCGGGTTTCCCAGTTTACTGTGTCAGGATTGCCCCTCACGTCATGATGACATGGATCTTCCTAAACCAGATTACGAAATTTCAAAAGAAGATTGGTATGTGAATCACTAGTTCTCGAGCTCGGTACCTG |
| **Vector-specific forward primer** | GGTGACACTATAGAACTCACC |
| **Vector-specific reverse primer** | CCTATATATGGATCCAATTTATGG |

1.2. **Column efficiency**

The efficiency of the desalting column in both the first and second desalting steps was evaluated using EGFP-containing proteoliposomes prepared according to the column's specified method (default buffer volume: 2.9 mL; sample load volume: 0.5 mL). The 10 fractions of 200 µL were collected after loading 150 µL of fluorescent proteoliposome, followed by the addition of the loaded sample volume (0.5 mL). Recovery of fluorescent proteoliposomes was quantified by fluorescent intensity, which correlated with protein recovery. The recovery yield was 58% for the first desalting step and 80% for the second step. The recovery values were subsequently used to determine the final dilution factor (×96) and protein concentration for accurate calculation of transport activity.

For the second desalting step, column efficiency to remove the external metabolite was further evaluated using the radioactive tracer [^3^H]-putrescine. This method provides higher sensitivity and accuracy due to the strong signal (CPM) generated by tritium, enabling detection at the picomole level. Desalting efficiency was assessed across the collected fractions, and complete removal of unincorporated tracer was observed within the elution range, indicating 100% efficiency.

**Supplementary Material Fig.1. a.** The EGFP-proteoliposome recovery % for the first desalting to remove unincorporated cold α-ketoglutaric acid or malate, **b.** The EGFP-proteoliposome recovery % for the second desalting to remove unincorporated radioactive α-ketoglutaric acid, and **c.** the desalting efficiency of removing radioactive tracers. The column efficiency was evaluated in duplicate (n=2), and the results are shown as the mean.

1.3. **Protein quantification using the BSA assay**

Proteoliposomes obtained after the washing step were resuspended in 500 µL of buffer and sonicated for 18 seconds to ensure uniform dispersion. An aliquot (100 µL) of proteoliposome suspension was mixed with an equal volume of 2X RIPA buffer and incubated at room temperature for 1 hour, with intermittent vortexing every 15 minutes to aid protein solubilization. The total protein concentration was then measured using a bovine serum albumin (BSA) standard curve according to the manufacturer's instructions. The protein concentrations for 150 µL of SLC25A11, ALC25A21, and AtDTC proteoliposomes were 0.053±0.002, 0.073±0.003, and 0.009±0.002 mg, respectively.

1.4. **Coomassie-stained SDS-PAGE**

Coomassie-stained SDS-PAGE was performed to evaluate the expression and apparent molecular mass of the reconstituted membrane transporters. Equal volumes (7.5 μL and 37.5 μL) of proteoliposome preparations containing AtDTC (0.93 μg/μL), SLC25A11 (0.92 μg/μL), or SLC25A21 (0.97 μg/μL) were analyzed alongside a blank liposome control. Samples were separated on a 10% SDS–polyacrylamide gel and stained overnight with Coomassie Blue R-250 solution (0.1% [w/v] R-250, 50% [v/v] methanol, 10% [v/v] acetic acid, and 40% water). The gel was then destained for 2 h using a destaining solution (50% [v/v] methanol, 40% water, and 10% [v/v] acetic acid), followed by overnight incubation in Milli-Q water. The gel was imaged using a ChemiDoc Touch Imaging System (Bio-Rad).

The predicted molecular masses were compared with the migration positions of the major bands observed in the corresponding sample lanes. SLC25A11 consists of 314 amino acids and has a calculated molecular mass of approximately 34.1 kDa, whereas SLC25A21 contains 299 amino acids with a calculated molecular mass of approximately 33.3 kDa. Thus, the relatively small difference in sequence length (15 amino acids) is consistent with the closely spaced electrophoretic migration expected for these two proteins. The prominent bands indicated by asterisks migrate within the approximately 30–35 kDa region of the molecular-weight standards, consistent with the predicted size range of these SLC25 transporters. The corresponding bands were substantially enriched in the transporter-containing samples compared with the surrounding protein background.

Importantly, the observed band position is consistent with the theoretical molecular mass, enrichment pattern, and subsequent functional characterization, which provides evidence of successful recovery of the recombinant transporters.

These results demonstrate that the preparations contained detectable transporter protein within the expected apparent molecular weight range and were suitable for subsequent reconstitution and transport activity measurements.

**Supplementary Material Fig. 2.** Coomassie-stained SDS-PAGE was performed to evaluate the expression and apparent molecular mass of AtDTC, SLC25A11, and SLC25A21.

1.5. **Calculation of the proteoliposome substrate rate of uptake (activity)**

The enzymatic activity of the SLC25A11, SLC25A21, and AtDTC using radiolabeled substrate (here, α-Ketoglutaric acid) was calculated with a slight modification from Zhifeng Chen, *et. al,* work ^1^:

$$Specific activity (\frac{CPM}{nmol})=\frac{Quantified CPM}{Specific nmol}$$

Where the standard CPM for 3.64 nmol α-Ketoglutaric acid was 585684 CPM.

The content of the transported substrate in the proteoliposomes was then calculated by:

$$Activity (\frac{\mathrm{nmol}}{mg of protein . min})=\frac{sample CPM \times dilution factor}{specific activity \times protein \left( mg \right)\times incubation time (\min)}$$

The protein concentration was measured after washing and resuspending in 500 µL buffer at the initial step before reconstituting into preloaded liposomes. The dilution factor was the dilution of the proteoliposome from the reconstitution process in the preloaded liposome, up to just before resuspending the proteoliposome into a liquid suspension, taking the column recovery rate into account.

1.6. **Size and concentration of proteoliposomes**

The size and concentration of particles were evaluated using IZON-N250 nanopore and calibration particles of 200 nm (CPC200).

**Supplementary Material Fig. 3.** **a.** shows the size distribution of proteoliposomes and liposomes without protein, and **b.** shows the concentration of proteoliposomes and liposomes without protein. Data shown as a mean n = 9 or n = 3, Tukey’s multiple comparison test, P-value ****< 0.0001, and ns = no significant.
